# Real Time Measures of Prestin Charge and Fluorescence during Plasma Membrane Trafficking Reveal Sub-Tetrameric Activity

**DOI:** 10.1371/journal.pone.0066078

**Published:** 2013-06-10

**Authors:** Shumin Bian, Dhasakumar Navaratnam, Joseph Santos-Sacchi

**Affiliations:** 1 Department of Surgery (Otolaryngology), Yale University School of Medicine, New Haven, Connecticut, United States of America; 2 Department of Neurobiology, Yale University School of Medicine, New Haven, Connecticut, United States of America; 3 Department of Neurology, Yale University School of Medicine, New Haven, Connecticut, United States of America; 4 Department of Cellular and Molecular Physiology, Yale University School of Medicine, New Haven, Connecticut, United States of America; University of South Florida, United States of America

## Abstract

Prestin (SLC26a5) is the outer hair cell integral membrane motor protein that drives cochlear amplification, and has been described as an obligate tetramer. We studied in real time the delivery of YFP-prestin to the plasma membrane of cells from a tetracycline-inducible cell line. Following the release of temperature block to reinstate trans Golgi network delivery of the integral membrane protein, we measured nonlinear capacitance (NLC) and membrane fluorescence during voltage clamp. Prestin was delivered exponentially to the plasma membrane with a time constant of less than 10 minutes, with both electrical and fluorescence methods showing high temporal correlation. However, based on disparity between estimates of prestin density derived from either fluorescence or NLC, we conclude that sub-tetrameric forms of prestin contribute to our electrical and fluorescence measures. Thus, in agreement with previous observations we find that functional prestin is not an *obligate* tetramer.

## Introduction

In altricial mammals, postnatal development of hearing requires a concerted assembly of key proteins in hair cells, for example prestin (SLC26a5) in the lateral membrane of the outer hair cell (OHC). The residence of prestin in the OHC plasma membrane can be assessed by nonlinear capacitance (NLC), a correlate of voltage-dependent conformational changes within the integral membrane protein [1], [2]. Since prestin is only measurable after membrane insertion, it has been used to gauge development of protein density; in rat and mouse, NLC magnitude rises from about day 3 to day 18 [3], [4]. In order to measure very early developmental characteristics of prestin, which proves difficult in native OHCs, we developed a tet-inducible cell line which expresses YFP-tagged prestin at levels well above that obtained from transient transfection methods [5]. Consequently, we were able to measure Boltzmann characteristics of prestin from about 2 to 48 hours, and found significant changes in these characteristics over time.

Newly synthesized integral membrane proteins are delivered to the plasma membrane from the trans Golgi network (TGN) [6], [7]. In the OHC, the TGN is localized to the apical cytoplasm, where vesicles possessing integral membrane proteins bud off, migrate and then fuse with the plasma membrane to effect delivery. Here we have taken advantage of the inducible nature of our cell line to monitor the delivery of prestin within minutes after the onset of TGN shuttling. We find that both NLC and membrane fluorescence provide equivalent estimates of delivery time. However, estimates of protein number based on NLC and fluorescence differ markedly. Using estimates of single particle fluorescence intensity, we interpret this discrepancy to indicate that prestin activity derives from sub-tetrameric forms of prestin.

## Materials and Methods

### Prestin cell lines and cell culture

Our tetracycline-inducible, highly-expressing monoclonal prestin HEK 293 cell lines were reported previously [5]. Cells were cultured in Dulbecco's modified Eagle's medium (DMEM) high glucose medium containing 50 U/ml each of penicillin and streptomycin, 10% fetal bovine serum at 37°C in a 5% CO_2_ incubator. 4 µg/ml of blasticidin and 130 µg/ml of zeocin were supplemented in the growth media to maintain prestin expression.

### Electrophysiology measurements

A Nikon Eclipse FNI upright microscope equipped with a 40× water immersion lens and a green fluorescent protein (GFP) UV light filter was used for cell observation. Whole cell patch-clamp measurements were made with an Axopatch 200B Integrating Patch clamp (Axon Instruments) and a Digidata 1322A A/D converter. An EXFO motorized manipulator by Burleigh (PCS-6000) was used for pipette manipulation. Unless otherwise specified, intracellular solution (pipette solution) contained (in mM) 110 KAsp, 10 NaCl, 10 KCl, 1.2 CaCl2, 1 MgCl2, 10 HEPES, and 5 EGTA, pH 7.28. Osmolarity was adjusted to 348±2 mosM using glucose, to be the same as the bath solution. Extracellular solution (bath solution) contained normal growth media plus 4 mM 4AP and 5 mM TEA, with pH pre-adjusted to 7.3. Initial experiments were done with 140 chloride intracellular solutions to maximize NLC measures; however, we switched to ∼20 mM solutions which we found to help stabilize recordings during temperature changes. 20 mM chloride is well above the K_d_ for prestin sensitivity (1–6 mM; [8]–[10]), so that NLC magnitudes were appreciably optimized for our observations. Glass pipettes were pulled using a P-2000 laser-heating pipette puller (Sutter Instruments). All pipettes had initial resistances of about 2.5 MΩ. Cell capacitance was measured under whole cell configuration using the jClamp software (Scisoft, CT; www.SciSoftCo.com).

Nonlinear capacitance was measured using a continuous high resolution (2.56 ms sampling) two-sine stimulus protocol (10 mV peak at both 390.6 and 781.2 Hz) superimposed onto the voltage ramp [11], [12]. When possible, capacitance traces were fit to the first derivative of a two-state Boltzmann function [2].
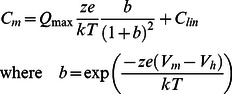
(1)Q_max_ is the maximum nonlinear charge moved, V_h_ is voltage at peak capacitance or equivalently, at half maximum charge transfer, V_m_ is membrane potential, z is valence, C_lin_ is linear membrane capacitance, e is electron charge, k is Boltzmann's constant, and T is absolute temperature.

### Fluorescence imaging

Images were taken with a SPOT RT Slider CCD camera cooled to – 5°C (Diagnostic Instruments, Inc.) with SPOT for Windows software control. General fluorescence exposure conditions are the following: GFP filter, 8 second exposure, 16× NDF, 40× water lens. Selection of ROI and fluorescence intensity integration were done using ImageJ software (NIH). The membrane region of interest determined from phase-contrast images where the membrane was clearly visible. The cell's shape was nearly spherical because it was lifted up from the perfusion chamber bottom during patch recording. The ROI was set to include the outline of the membrane, the width of the ROI determined from the need to form limiting circular outlines that included deviations of the membrane from true a true sphere.

### Bleach correction

Since the fluorescence illumination area is over 1000 times larger than a 2D cell area, we can assume the bleaching factor for the soma of the cell is the same as for the membrane. Our method to correct for bleaching is based on a two compartment model, namely, soma (Golgi with associated transport vesicles) and plasma membrane. Theoretically, since cells are under patch clamp, loss of prestin from the cell into the patch pipette is possible, but we predict quite remote. First, prestin molecules are not freely floating in the cytosol, but are within the lipid bilayer of Golgi-derived transport vesicles, expected to be roughly 75 nm in diameter. In a two compartmental study of vesicle transport, Klann et al [13] estimate that 50 nm vesicles possess diffusion coefficients that can range from .04e^−7^ to .0016e^−7^ cm^2^/s. Of course, a 75 nm vesicle would have even poorer diffusion. Nevertheless, given these coefficients and simply taking Pusch and Neher's (1998) formula (eq. 17) for diffusional washout via a patch pipette, we calculate based on our pipette access resistance of 7.5 MΩ, and our cell size (_∼_12 pF), that the range in washout tau would be from 710–17766 minutes. This range is far greater than the 6–8 min tau of prestin delivery that we find. The slower tau takes into account the well-known utilization of cytoskeletal elements in guiding vesicle delivery to the membrane, thereby reducing the possibility of freely diffusible vesicles due to binding (see Schroer and Schatz [14]). We are confident that our observations can be treated as a two compartment problem.

At any given time t, the integrated average fluorescence intensity deceases due to bleaching for each pixel 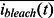 can be applied both intracellularly and on the membrane. As a first order approximation, assuming the processes that happen within the 2D image can largely represent the 3D cell, we have
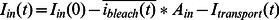
(2)

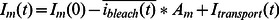
(3)where 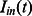 is the integrated intracellular fluorescence density at time *t*; 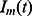 is the integrated membrane fluorescence density at time *t*; 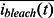 is the integrated fluorescence decrease per pixel due to bleaching; 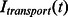 is the integrated fluorescence change at time *t* due to trafficking from intracellular space to the membrane; 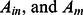 are the integrated intracellular area and membrane area respectively, which are also time dependent taking changes in cell shape into consideration. From these two equations, we can derive the following two:
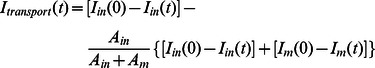
(4)

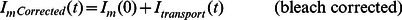
(5)where 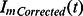 is the bleach-corrected integrated membrane fluorescence at time *t*. Membrane fluorescence values were scaled by 20 to estimate whole cell fluorescence, so that it could be compared to NLC (derived from whole cell measures). The scaling factor was based on a spherical cell's membrane surface area for a 20 µm diameter cell and a depth of field of 2 µm. Thus, at 18 minutes after temperature block we found a membrane intensity of 3564040 AU, which after scaling was 71280800 AU. Membrane fluorescence of the spherical cell outside the depth of field could slightly contribute to intracellular values, possibly accounting for the slight difference in slopes (or τ) of fluorescence vs NLC increase after temperature unblock.

### Temperature block (T-block) experiments

Freshly plated tet-inducible HEK293 cells on cover slips were allowed to stabilize for 3 hours at 37°C. The cells were then treated with 1.0 µg/ml tetracycline for 60 min at room T (21°C) for protein synthesis before transferred to a bath solution consisting of normal growth media plus tetracycline supplemented with 4 mM 4-AP and 5 mM TEA for whole-cell patch clamp. Upon establishment of whole-cell configuration, the cell was lifted up slightly from the coverslip using the patching pipette to avoid crushing onto the coverslip during heating. After reference NLC and fluorescence measures were made, the release of temperature block was initiated by increasing the temperature of the bath solution. Temperature control was achieved by using a HCC-100A Heating/Cooling Bath Temperature Controller (Cornerstone series by Dagan) equipped with a Block and a Bath thermal sensing probes fitted on a HE-101 electrical thermal stage. Custom-modified glass bottom 35 mm microwell dishes (MatTek, MA) were used for plating cells and for fitting into the thermal stage. Bath solution volume was 1.6 ml, optimized for heating rate and taking into consideration of vaporization. Heating rate was pre-adjusted to be around 1°C per minute (up to a maximum of 37°C) unless otherwise specified, so as not to shock the cells. Simultaneous monitoring of NLC and fluorescence was done. Capacitance traces were taken every 2 minutes using an automated script sampling program inside jClamp, and phase-contrast images and YFP fluorescence photos were taken every two minutes, synchronized with NLC measurements.

### Diffraction-limited fluorescence single particle analysis

Diffraction-limited fluorescence single particle analysis was done using similar approaches as reported in Hallworth and Nichols [15], namely by using membrane preparations with standard illumination. Tet-inducible prestin-expressing HEK cells growing on cover slips were incubated with 1.0 µg/ml tetracycline for 10 hours at 37°C incubator, then lysed by hypo-osmotic solution consisting of 4 mM MOPS pH6.2 by KOH and 30 mM KCl at 4°C for 30 min. After washing vigorously with cold lysis buffer, the coverslip was transferred to PBS buffer at room temperature, and membrane pieces observed with fluorescence microscope under the same condition as for monitoring trafficking. For diffraction-limited single particle picking, uniform box size 4×4 square pixels corresponding to 760×760 nm^2^ area were used, very close to the box size used by Hallworth et al. (750×750 nm^2^). Larger brighter spots that extended beyond the ROI, and undersized spots that visually lacked fluorescence in four pixels were excluded. Integrated fluorescence of each spot was quantified after global background correction.

## Results

### NLC measurements can detect newly synthesized prestin delivery to the plasma membrane in real-time

**Figure 1:**
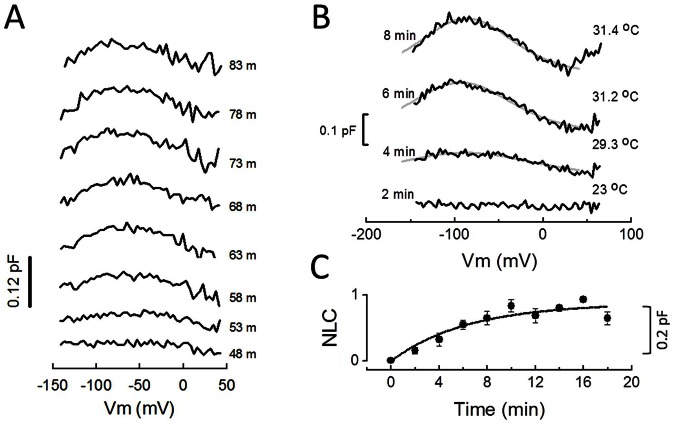
Prestin delivery to the cell surface can be monitored in real time by NLC measurements. **A**) For initial experiments, tet-inducible HEK293 cells were incubated with 1.0 µg/ml tetracycline for 30 min at 37°C prior to whole cell recording in normal growth media plus tetracycline at room temperature (24°C). In this case, intracellular (pipette) solution was (in mM) 128 KCl, 5 MgCl_2_, 0.5 CaCl_2_, 5 EGTA, pH7.28. Cell membrane capacitance traces were recorded every 5 minutes, and the time stamp on traces indicates the time following tetracycline. Prestin NLC clearly develops over time. All traces are subtracted with the 43 minute trace. **B**) Synchronized transient delivery of newly synthesized prestin to the plasma membrane from TGN occurs after release of T-block. Freshly plated tet-inducible HEK293 cells on cover slips were allowed to settle for 3 hours at 37°C before incubation with 1.0 µg/ml tetracycline for 60 min at 21°C for protein synthesis. Cells were then transferred to a bath solution composed of normal growth media plus tetracycline supplemented with 4 mM 4-AP and 5 mM TEA for whole-cell patch clamp. Intracellular (pipette) solution is given in Methods. Cell membrane capacitance traces were taken every 2 minutes, and the release of temperature block was initiated by increasing the temperature of the bath solution after whole-cell configuration was established and the cell was detached using the pipette. In order to emphasize the increase over time, NLC traces are subtracted with 0 time trace. Prestin trafficking from trans-Golgi network to the plasma membrane occurs quickly. **C**) Normalized NLC change during transient delivery of prestin to the plasma membrane following release of T-block. NLC changes either in Peak NLC or in fitted Q_max_ were averaged (+/− se, n = 3–8 cells) for each time point as measured in **B**. Normalized NLC data were fit with a single exponential, ***τ*** being 6.4 minutes. The plateau likely reflects the depletion of membrane-bound prestin molecules from the trans-Golgi complex.

We have previously measured NLC increases for tet-inducible prestin-expressing cell lines from 2 hours to 72 hours [5]. Our tet-inducible system enables us to characterize early maturational events of the motor protein prestin that cannot be made in native outer hair cells, due to the difficulty in isolating and recording OHCs at very early stages of development. Given the potential of our system, we asked whether we can monitor prestin as it is being synthesized and transported to the cell surface membrane in real time. To address this question, we tested cells after half hour incubation with tetracycline at 37°C. NLC starts to appear at 10–60 minutes after induction (**Figure 1a**). The continued growth of peak NLC near −80 mV from individual cells indicates we can monitor real time prestin delivery to the cell surface.

### Measurement of synchronized delivery of newly synthesized prestin to the plasma membrane from TGN

Though we can monitor the delivery of prestin to the plasma membrane with NLC measures, the initiation of delivery among cells varies widely, necessitating prolonged whole-cell recording. Due to these technical difficulties, we sought a method to synchronize delivery to the membrane, and limit recording time. It is well established that membrane-bound proteins can be synthesized on ER at room temperature (20°C), but these newly synthesized protein molecules are trapped in the TGN until release of this temperature block (T-block) by an increase of environment temperature [6], [7], [16]. We took advantage of this phenomenon (see Methods) and were able to monitor synchronized prestin delivery to the cell membrane after release of T-block, as shown in **Figure 1b**. Within minutes after the release of T-block, NLC started to increase. The maximum amplitude of prestin peak NLC increase varied from cell to cell, averaging 0.195 +/−0.051 pF at 12 minutes (mean +/− se; n = 7). In cells where Boltzmann fits were possible, *z* was 0.49+/−0.043 at 12 minutes (n = 4), similar to measures we previously made from 2–4 hours after induction [5]. The time course of trafficking from TGN to the plasma membrane after release of T-block could be fit with a single exponential representing the kinetics of a two compartment transfer, as shown in **Figure 1c**. The time constant (τ) of 6.4 minutes is similar to some other membrane proteins such as AChR [7].

### Prestin-YFP fluorescence in the membrane correlates with development of NLC

**Figure 2:**
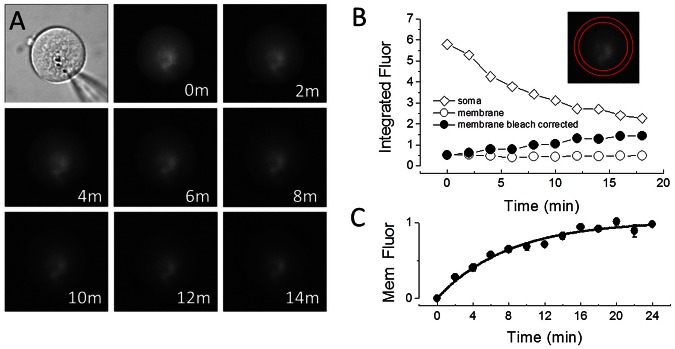
Prestin delivery to the cell surface can be monitored in real time by fluorescence measurements. **A**) A phase-contrast image and consecutive YFP fluorescence photos taken every two minutes, simultaneous with NLC measurements as in **Figure 1**. Exposure conditions: SPOT CCD camera, 8 second exposure, 16× NDF, 40× lens. While there is a clear photo-bleaching effect intracellularly, the fluorescence intensity on the cell membrane is kept near constant, indicating an added component from trafficking. **B**) Measurement of intracellular integrated fluorescence density (open diamonds) and that of the membrane (open circles) as indicated by the ROI in the photo insert. Following bleach correction according to Equations (2–5) membrane fluorescence shows an increase over time (filled circles). **C**) Membrane fluorescence changes for each time point as measured above were averaged (+/− se, n = 14 cells) and normalized. Normalized NLC data were fit with a single exponential, ***τ*** being 7.9 minutes. As with NLC, the plateau likely reflects the depletion of membrane-bound YFP-prestin molecules from the trans-Golgi complex.

We would like to develop an alternative approach to monitor real time prestin trafficking to and from the membrane that does not rely on patch clamp recording. With NLC measurement as a simultaneous reference, we assessed changes in YFP fluorescence at the membrane as an indicator of prestin trafficking. **Figure 2a** shows a whole-cell patched HEK cell and a series of 2D fluorescence images taken under low-light conditions using a SPOT CCD camera following release of T-block; NLC measurements were simultaneously made. After uniform background correction in ImageJ, membrane fluorescence and intracellular fluorescence were integrated by drawing ROI for each area, as shown in **Figure 2b**. While intracellular fluorescence (open diamonds) decreases exponentially over time due to bleaching, membrane fluorescence (open circles) remains roughly constant. Bleach-corrected integrated membrane fluorescence (see Methods) shows a clear increase in fluorescence due to membrane trafficking (filled circles).

**Figure 3:**
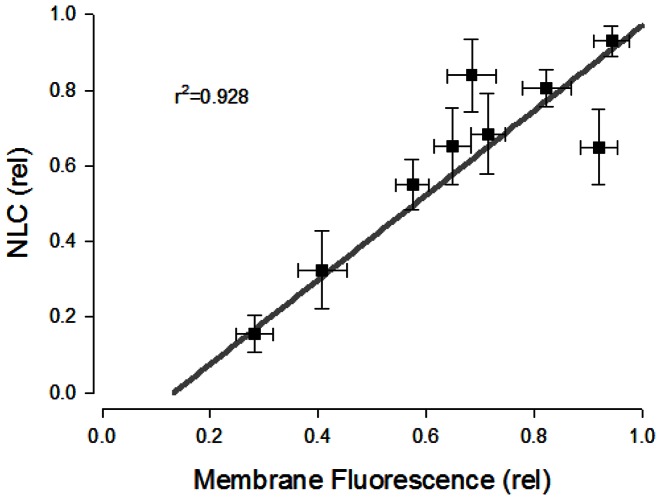
Correlation of membrane surface fluorescence and NLC. Data from **Fig. 1C** and **Fig. 2C** are plotted against each other. The high correlation (r^2^ = 0.983) between the two confirms that either can be used to monitor kinetics of prestin delivery to the plasma membrane following release from T-block.

After release of T-block, normalized average membrane fluorescence (bleach-corrected) increases over the course of minutes (**Figure 2c**), and can also be fit by an exponential equation with a τ of 7.9 minutes, similar to that of NLC. Indeed, the correlation of NLC and membrane fluorescence is high (**Figure 3**), confirming the successful use of fluorescence to measure YFP-tagged prestin delivery to the membrane.

### How many prestin molecules are trafficked from TGN to the plasma membrane following T-block?

The membrane fluorescence measures we have made allow us to estimate numbers of prestin molecules housed in the TGN and subsequently delivered to the plasma membrane. In order to utilize membrane fluorescence data to arrive at quantitative prestin delivery, we must estimate the unit fluorescence value of prestin molecules. In their study on the oligomeric state of prestin, Hallworth and Nichols [15] used eGFP-tagged prestin fluorescence imaging to observe diffraction-limited fluorescent spots consistent in size with single molecules. Step-wise fluorescence bleaching of these single molecules fits well with a binomial model of prestin oligomerization, which implies prestin is an obligate tetramer. This approach was originally developed for analysis of other membrane proteins [17]. Using this approach (see Methods) and taking the size of single prestin molecules from Hallworth and Nichols as a reference, we measured single molecule fluorescence intensity under the same measurement conditions as in our integrated membrane fluorescence measures to derive the number of prestin motors trafficked to the membrane. As with Hallworth and Nichols [15], we assume that prestin transfected cells are an appropriate model of OHCs for determination of oligomer state, and that preparative techniques did not alter oligomer state.

**Figure 4:**
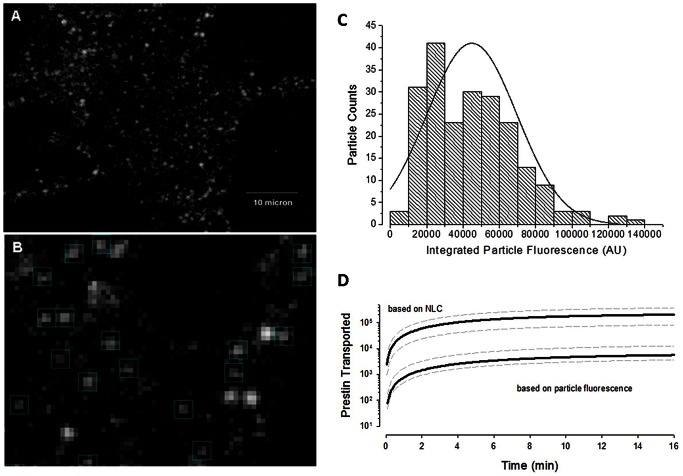
Estimates of prestin monomers deleiverd to the membrane following release from T-block. **A**) Prestin molecules transported to cell membrane visualized as diffraction-limited fluorescence spots consistent in size with single molecules (Hallworth and Nichols, J Neurophysiology, 2011). Membrane pieces observed with fluorescent microscope under the same condition as described in **Fig. 2** for monitoring trafficking. Scale bar 10 µm. **B**) Enlarged view for particle picking for fluorescence quantification. Uniform box size 4×4 square pixels corresponding to 760×760 nm^2^ area, very close to the box size used by Hallworth et al. (750×750 nm^2^). Bright, larger spots likely representing multiple particles were excluded. Integrated fluorescence of each spot was quantified after global background correction, the same way as done in **Fig. 2**. **C**) Particle intensity distribution. Data were automatically binned and fitted in Origin. The fact that the regression line does not pass through the origin may indicate that non-functional fluorescent monomers contributed to membrane fluorescence. **D**) Estimates of monomer deposition into the membrane obtained using NLC or fluorescence measures (see Methods). Grey dotted lines depict +/− standard deviation. Note discrepancy, with NLC estimates giving greater than an order of magnitude increase in numbers of monomers.

**Figure 4a** shows one of the micrographs of diffraction-limited fluorescence spots resulting from prestin-YFP delivery to the membrane after 10 hours. We used 10 hours post induction to obtain sufficient material for analysis. **Figure 4b** shows an enlarged area from which we hand-picked prestin single particles according to dimensions used by Hallworth and Nichols [15]. Only those particles with a size of about 750 nm were picked with a box size of 4×4 square pixels corresponding to 760×760 nm^2^. Larger brighter spots that extended beyond the ROI, and undersized spots that visually lacked fluorescence in four pixels were excluded. The average integrated particle fluorescence intensity is 44793+/−24846 AU (mean +/− sd; n = 211 samples). A plot of the intensity distribution is shown in **Fig. 4c**. Taking this number as a tetramer, we converted the membrane fluorescence increase (71280800 AU @ 18 min) to the number of prestin molecules inserted into the membrane, as shown in **Figure 4d**. On average, about 6000 prestin monomers (4*1500 tetramers) are inserted into the membrane after the release of T-block.

Peak NLC measures also provide estimates of the number of prestin molecules delivered from the TGN to the plasma membrane. Based on 0.195 pF peak NLC and a ***z*** value of 0.49, total prestin charge moved (Q_max_) would be about 40.6+/−26.0 fC (mean +/sd; 4kT/ze * NLC [18]). We have previously studied the effect of temperature on mature prestin in HEK cells and found that while the operating voltage range of prestin is very susceptible to temperature, Q_max_ is not [19]. At a unit electron charge, widely acknowledged as prestin's approximate unit voltage sensor charge [1], [2], [20], total charge movement would correspond to 253,000+/−163,000 prestin molecules (mean +/− sd). The development over time is also illustrated in **Figure 4d**.

In comparing the disparate monomer estimates based on membrane capacitance to those derived from NLC, we note that methodology could be influential. For example, using smaller values for prestin's unit charge would increase plasma membrane monomer estimates based on NLC. Our choice of 1 unit electron charge is therefore conservative, tending to diminish the disparity between monomer estimates based on NLC and fluorescence. We also note that it may be possible that non-functional prestin motors (lacking NLC) are inserted into the membrane and contribute to membrane fluorescence (see **Fig. 4c**). The presence of these non-functional monomers would tend to increase fluorescence based estimates of plasma membrane monomers, also tending to diminish the disparity between monomer estimates based on NLC and fluorescence.

## Discussion

Our tet-inducible prestin cell line provides specific nonlinear charge (sensor charge/linear capacitance) values of about 20 pC/pF after 24 hrs, far greater than transient transfection can provide [5]. This efficiency allowed us to monitor, real-time, the NLC associated with prestin delivery and insertion into the plasma membrane. Nevertheless, the variability in the timing of expression following tetracycline induction led us to use methodology to synchronize trafficking for quantitative assessment. Thus, we used well established temperature-block methodology [6], [7], [16] to synchronize delivery of prestin to the plasma membrane. Prestin was synthesized on ER at 20°C, but it was trapped in the TGN until release of this temperature block.

### Time course of prestin delivery from TGN to plasma membrane

By utilizing temperature block/unblock to synchronize prestin delivery from the TGN to the plasma membrane we were able to measure cargo delivery having an exponential rise, with τ of 6.4 minutes. This timing was confirmed by measuring the development of membrane fluorescence due to prestin-YFP delivery, τ in this case being 7.9 minutes. Indeed, the correlation between membrane fluorescence and NLC was very high, indicating that either methodology could be used to study trafficking of prestin. This is important, since membrane fluorescence measures are far simpler and more efficient than whole cell patch clamp. The time course of delivery that we find is similar to that found in other studies on integral membrane proteins [7]. The ability to monitor prestin delivery to the plasma membrane will no doubt aide in understanding the cell biology of this important protein. In fact, our observations allow us to focus on the timely question of prestin oligomerization.

### What is the functional unit of prestin?

The simple 2-state Boltzmann models that are used for the characterization of prestin's charge movement assume that prestin functions as an independent species, with valance close to 1 [1], [2]. Additionally, the blocking effect of salicylate on prestin, whose site of action is intracellular, has a Hill coefficient close to 1 [21], implying a lack of cooperativity. Likewise, the Hill coefficient of chloride effects on prestin is also near 1 [8], [9]. These data may imply that monomeric prestin is functional.

Nevertheless, we and others have since provided evidence that prestin additionally functions as a dimer [5], [22], [23]. For example, we found that prestin valence ***z***, which informs about the protein's unitary charge or voltage sensitivity, increased from 0.5 to 0.8 over the course of 4 hours as prestin accumulates in the plasma membrane following tet induction [5]. Having identified multimeric forms of prestin after 4 hours, we speculated that changes in ***z*** could have resulted from some maturational event, e.g., multimerization. The self-association of prestin monomers has been linked to their residence in cholesterol containing lipid rafts in HEK cell plasma membranes [24], [25]. Cholesterol was found to favor dimer formations. Recently, suggestions for a tetrameric structure have been proposed [15], [26]. Indeed, Hallworth and Nichols used step-wise fluorescence bleaching of single prestin molecules to suggest that prestin is an obligate tetramer [15].

Here we have used similar methodology to Hallworth and Nichols to determine the fluorescence intensity of assumed tetramers of prestin to estimate the number of prestin molecules reaching the plasma membrane at steady state after temperature unblock. We found that our florescence methodology, whose kinetic estimates of YFP-prestin delivery are in quantitative agreement with measures of prestin charge delivery, provides over an order of magnitude underestimate of prestin monomer numbers relative to numbers derived from charge measures.

The estimate of prestin number based on charge has a long history and we are generally confident in this approach. Indeed, we calculate that about five hundred TGN-derived vesicles, about 75 nm in diameter [6], with a density of 5000 monomers per µm^2^ were required to account for our NLC measures. Similarly, Andreose et al. found that TGN-derived vesicles delivered ACh receptors at that high density [7]. We conclude that our presumed tetrameric single particle intensity measures actually arise from a higher order of oligomer, or more likely that fluorescent particles of smaller size, representing sub-tetrameric structures, contribute to the total fluorescence and NLC that we measure. Consequently, our present data and previous data of others indicated that functional prestin is not an *obligate* tetramer.
